# Nanoparticle-delivered antimicrobials for targeted suppression of bacterial wilt in peanut

**DOI:** 10.3389/fmicb.2025.1740992

**Published:** 2026-01-14

**Authors:** Yohannes Gelaye, Huaiyong Luo

**Affiliations:** 1 Key Laboratory of Biology and Genetic Improvement of Oil Crops, Ministry of Agriculture, Oil Crops Research Institute of the Chinese Academy of Agricultural Sciences (CAAS), Wuhan, China; 2 Department of Horticulture, College of Agriculture and Natural Resources, Debre Markos University, Debre Markos, Amhara, Ethiopia; 3 National Nanfan Research Institute (Sanya), Chinese Academy of Agricultural Sciences, Sanya, China

**Keywords:** antimicrobial delivery systems, bacterial wilt, Peanut, NPs, *Ralstonia solanacearum*

## Abstract

Bacterial wilt caused by *Ralstonia solanacearum* is a major constraint to global peanut production, leading to serious yield and economic losses, particularly in tropical and subtropical regions. The disease reduces plant vigor, pod development, and overall productivity, posing a significant threat to food security and farmer income. Conventional control methods, including crop rotation, resistant varieties, soil amendments and chemicals, remain inconsistent and often provide limited long-term effectiveness. This inconsistency is due to the pathogen’s broad host range, prolonged soil survival, and high genetic adaptability, which enable rapid spread and persistence. These challenges indicate the need for sustainable alternatives that are effective and environmentally sound. Nanoparticle-based antimicrobial delivery systems have emerged as a promising strategy because of their precision targeting, improved stability, enhanced bioavailability, and controlled release of active agents. Key nanomaterial design parameters, including composition, size, surface functionalization, and carrier efficiency, critically influence antimicrobial activity against *R. solanacearum*. These characteristics affect interactions with bacterial cells and plant tissues. Major mechanisms of pathogen suppression involve membrane disruption, metabolic interference, oxidative stress generation, and induction of plant systemic resistance. Environmental aspects, such as nanoparticle fate, bioaccumulation, persistence in soil, and ecotoxicological risks, must also be considered to ensure ecological safety and sustainability. Integrating nanotechnology with plant breeding and biocontrol strategies can promote resilient and eco-friendly peanut production. Nanoparticle-enabled disease management offers a transformative approach for mitigating bacterial wilt while strengthening sustainable crop protection systems worldwide. Policy support and responsible innovation will accelerate the safe adoption of these technologies in the field.

## Highlights

- Bacterial wilt severely constrains sustainable peanut production worldwide.
- NPs enable targeted and efficient suppression of *R. solanacearum*.
- Metal and silica NPs disrupt pathogens and enhance host immunity.
- Silica NPs prime antioxidant systems and systemic acquired resistance.
- Nano-delivery systems improve antimicrobial stability, precision, and efficacy.

## 1 Introduction

Peanut (*Arachis hypogaea* L.) is one of the most significant oilseed and legume crops worldwide, serving as a vital source of edible oil, high-quality protein, and essential micronutrients (Arya et al., 2016; Bonku and Yu, 2020). Its cultivation supports millions of smallholder farmers, particularly in tropical and subtropical regions of Asia, Africa, and South America (Raza et al., 2024). Despite its economic and nutritional importance, peanut production faces severe challenges from bacterial wilt, a soil-borne vascular disease caused by *Ralstonia solanacearum* (*R. solanacearum*) (Yang et al., 2022). The pathogen invades plant roots and colonizes the vascular system, leading to rapid wilting and eventual plant death. In heavily infested fields, yield losses can exceed 80%, posing a critical threat to food security and farmer livelihoods (Pramesti and Umali, 2023).

*Ralstonia solanacearum* is a genetically diverse species composed of multiple races that infect distinct crops and cause severe yield losses worldwide. Race 1 has a broad host range and induces bacterial wilt in solanaceous crops such as tomato, potato, pepper, and peanut, often resulting in losses exceeding 70% (Yahiaoui et al., 2017). Whereas Race 2 causes Moko disease in banana and plantain, Race 3 is responsible for brown rot of potato and tomato in cooler regions, and Race 4 primarily affects ginger, leading to rhizome rot and substantial production decline. The pathogen’s vascular colonization and soil persistence limit the effectiveness of conventional control measures (Pardo et al., 2019; Champoiseau et al., 2009). Recent evidence indicates that Nanoparticles (NPs)-based strategies can effectively suppress *R. solanacearum* by enhancing antimicrobial delivery, disrupting bacterial cells, and activating plant defense responses (Cheng et al., 2020; Dilbar et al., 2023). Metal and metal-oxide NPs, including silver, zinc oxide, copper oxide, and silica, have shown promising disease-reduction effects in crops such as tomato, potato, ginger, and peanut, indicating the potential of nanotechnology as a versatile and innovative approach for mitigating *R. solanacearum*–induced crop losses (El-Lakkany et al., 2025; Khan et al., 2021).

In addition to direct antimicrobial effects, silica (SiO₂) NPs act as defense elicitors by enhancing antioxidant enzyme activities, including superoxide dismutase (SOD), catalase (CAT), peroxidase (POD), and ascorbate peroxidase (APX), thereby mitigating pathogen-induced oxidative stress (Wang et al., 2022; Li et al., 2025). SiO₂ NP application also elevates salicylic acid (SA) levels and upregulates defense-related genes, leading to the activation of systemic acquired resistance (SAR) (Deng et al., 2024). Hence, this immune-priming capacity states the dual role of NPs in combining pathogen suppression with host defense reinforcement against *R. solanacearum*.

Traditional management strategies for bacterial wilt such as crop rotation, the use of resistant cultivars, soil solarization, and chemical bactericides have shown inconsistent and often unsatisfactory outcomes (Mehan et al., 1992; Bahmani et al., 2021). This ineffectiveness arises from the remarkable adaptability of *R. solanacearum*, its wide host range, and its ability to persist in soil and water for extended periods. Moreover, the indiscriminate use of chemical agents contributes to environmental contamination, microbial resistance, and reduced soil health, thereby undermining long-term disease management efforts (Muñoz-Bautista et al., 2025). Consequently, the search for innovative, effective, and environmentally sustainable solutions has become an urgent priority in peanut disease management research.

In recent years, nanotechnology has emerged as a transformative tool in agricultural disease control. NPs possess unique physicochemical properties such as high surface area, tunable reactivity, and controlled release potential that enhance the delivery and efficacy of antimicrobial agents (Nedyalkova et al., 2025). Hence, when used for plant protection, NPs can facilitate targeted delivery to infection sites, improve the stability of active compounds, and minimize non-target effects. Several studies have demonstrated that metal and metal oxide NPs, polymer-based nanocarriers, and biogenic NPs exhibit significant antimicrobial activity against plant pathogens, including *R. solanacearum* (El-Lakkany et al., 2025). These developments open new avenues for designing efficient NP-mediated strategies for controlling bacterial wilt in peanuts. Therefore, this critical review aims to provide a comprehensive evaluation of the current progress, underlying mechanisms, and future prospects of NP-based antimicrobial delivery systems for the effective management of bacterial wilt in peanut.

## 2 Design and functionalization of NPs for targeted delivery

NP-based antimicrobial delivery systems have attracted growing attention as innovative tools for plant disease management, particularly for challenging soil-borne pathogens such as *R. solanacearum*. Varieties of NP types have been investigated for their antimicrobial potential, each offering distinct physicochemical and biological advantages (Wassie and Yemata, 2025; Spirescu et al., 2021). Metallic NPs such as silver (Ag), copper oxide (CuO), and zinc oxide (ZnO) exhibit strong bactericidal properties through mechanisms including membrane disruption, reactive oxygen species (ROS) generation, and interference with cellular enzymes (Franco et al., 2022; Parvin et al., 2025). Polymeric and lipid-based NPs, on the other hand, serve as biocompatible carriers capable of encapsulating both hydrophilic and hydrophobic antimicrobials, ensuring controlled release and enhanced bioavailability (Bhardwaj and Jangde, 2023; Lu et al., 2021). Silica and chitosan NPs contribute additional functionalities such that silica provides structural stability and a porous matrix for gradual release, while chitosan exhibits inherent antimicrobial and plant growth-promoting properties, making it particularly valuable for sustainable crop protection (Benkő et al., 2025; Ingle et al., 2022).

Metal organic frameworks (MOFs) are characterized by their high porosity, tunable pore sizes, large surface areas, and adjustable metal ligand chemistry, making them particularly suitable for controlled antimicrobial delivery. Recent studies demonstrate that MOFs, especially those based on metals such as zinc, copper, and iron, exhibit intrinsic antibacterial activity or can serve as efficient carriers for antimicrobial agents, including antibiotics, metal ions, and plant-derived bioactives (Kermanshahi and Akhbari, 2024). Their sustained-release behavior and responsiveness to environmental stimuli (e.g., pH or redox conditions) are particularly relevant for soil-borne pathogens such as *R. solanacearum*, which colonize the rhizosphere and vascular tissues.

Moreover, MOFs have been shown to reduce premature degradation of antimicrobial compounds in soil environments while enhancing localized delivery near infection sites (Livesey et al., 2023). Their potential to minimize off-target toxicity further supports their relevance in sustainable plant disease management.

Functionalization of NP surfaces represents a critical advancement in enhancing their efficiency and specificity. Surface modifications with agents such as polyethylene glycol (PEG), biopolymers, or plant-derived ligands not only improve NP stability under environmental conditions but also facilitate targeted interaction with plant root tissues and vascular systems (Shi et al., 2021; Kaymaz et al., 2023). These functional coatings can reduce aggregation, prolong NP persistence in soil, and improve translocation within plant tissues. Moreover, bio-inspired functionalization using natural compounds from peanut or associated rhizosphere microorganisms offers opportunities to align nanotechnology with ecological compatibility, potentially reducing phytotoxicity and enhancing selective uptake (Xu et al., 2021; Leng et al., 2023).

Equally important are controlled and targeted release mechanisms, which are being increasingly incorporated into NP formulations. Smart delivery systems that respond to specific environmental cues such as pH changes, pathogen-secreted metabolites, or enzymatic activity enable localized and on-demand release of antimicrobials at infection sites (Li et al., 2022). This precision delivery reduces the amount of active agent required, minimizes off-target effects, and enhances disease suppression efficiency. Moreover, the integration of responsive materials into NP systems signifies a significant step toward precision agriculture, where antimicrobial interventions can be both effective and environmentally sustainable (Zaman et al., 2025; Yadav et al., 2025).

**Figure 1:**
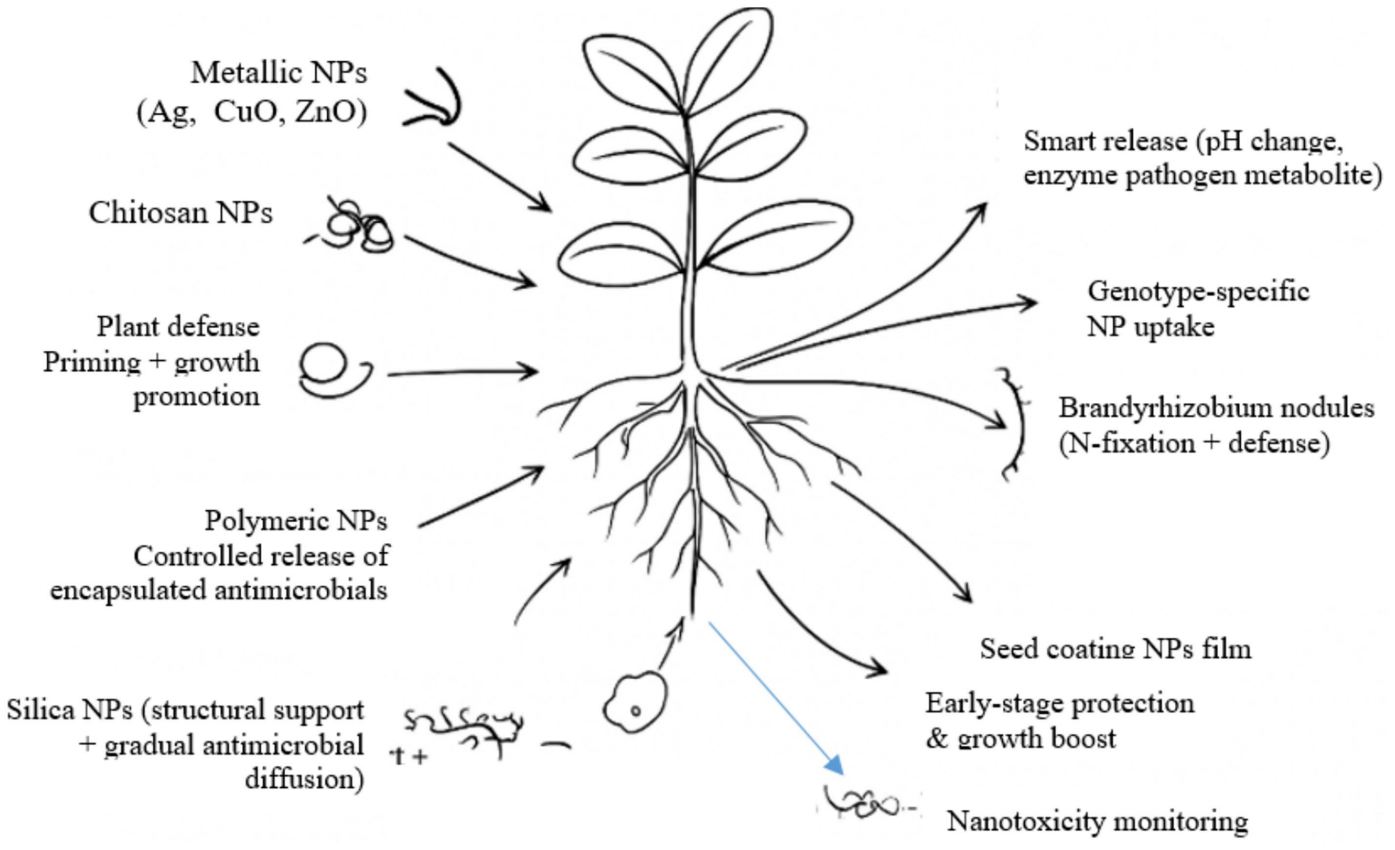
Nano-enabled framework for peanut *Ralstonia solanacearum* management.

Despite these promising developments, a major limitation remains the scarcity of peanut-specific studies. Most existing research on NP-mediated management of *R. solanacearum* has been conducted in solanaceous crops like tomato and potato, with limited direct application to peanut. Given the unique physiology of peanut roots, nodulation patterns, and soil-plant-microbe interactions, extrapolating results from other crops may not yield reliable outcomes. Hence, there is a pressing need for peanut-centric studies that optimize NP-type, dosage, and delivery strategy, while evaluating long-term impacts on soil health and beneficial microbiota. In general, the framework links multiple nanoparticle types with peanut physiological and microbial systems to achieve targeted suppression of *R. solanacearum* (Figure 1). It also indicates how functionalized and smart nanocarriers enhance antimicrobial efficiency, promote root defense activation, and maintain rhizosphere ecological balance.

## 3 Mechanisms of action beyond direct antimicrobial effects

NPs direct antimicrobial activity is primarily attributed to the release of bioactive metal ions, generation of ROS, and disruption of microbial cell structures (Liu et al., 2024; Jiang et al., 2024). ROS production induces oxidative stress that damages nucleic acids, proteins, and lipids, ultimately leading to cell death (Redza-Dutordoir and Averill-Bates, 2016). Then, the nanoscale size of these particles facilitates intimate contact with microbial cells, enabling efficient pathogen inhibition even at low concentrations. This mechanism provides an advantage over conventional bactericides, which often fail due to reduced bioavailability.

Beyond direct toxicity, certain NPs also activate host defense responses, thereby strengthening the plant’s innate immunity (Masood et al., 2024). Si, ZnO, and CuO NPs have been shown to enhance the expression of pathogenesis-related (PR) proteins, enhance antioxidant enzyme activity, and stimulate phenolic compound biosynthesis in plants (Verma et al., 2024; Essa et al., 2025). These physiological responses contribute to the reinforcement of cell walls and detoxification of ROS, creating a more resistant host environment against *R. solanacearum* infection. In peanut, such NP-induced priming could provide durable resistance by enabling the plant to respond more rapidly and effectively to pathogen attack (Deng et al., 2024; Kumar et al., 2025).

A further dimension of NP action involves modulation of the rhizosphere microbiome and by influencing microbial community composition and diversity; NPs can suppress pathogenic populations indirectly while favoring beneficial microorganisms such as *Bacillus* and *Pseudomonas* species (Zhang et al., 2024; de Moraes et al., 2021). This ecological rebalancing of the rhizosphere may enhance soil health and improve plant resilience. However, the long-term stability and reversibility of these microbial shifts remain insufficiently explored, particularly in peanut agroecosystems where nitrogen-fixing symbioses play a critical role in productivity.

**Figure 2:**
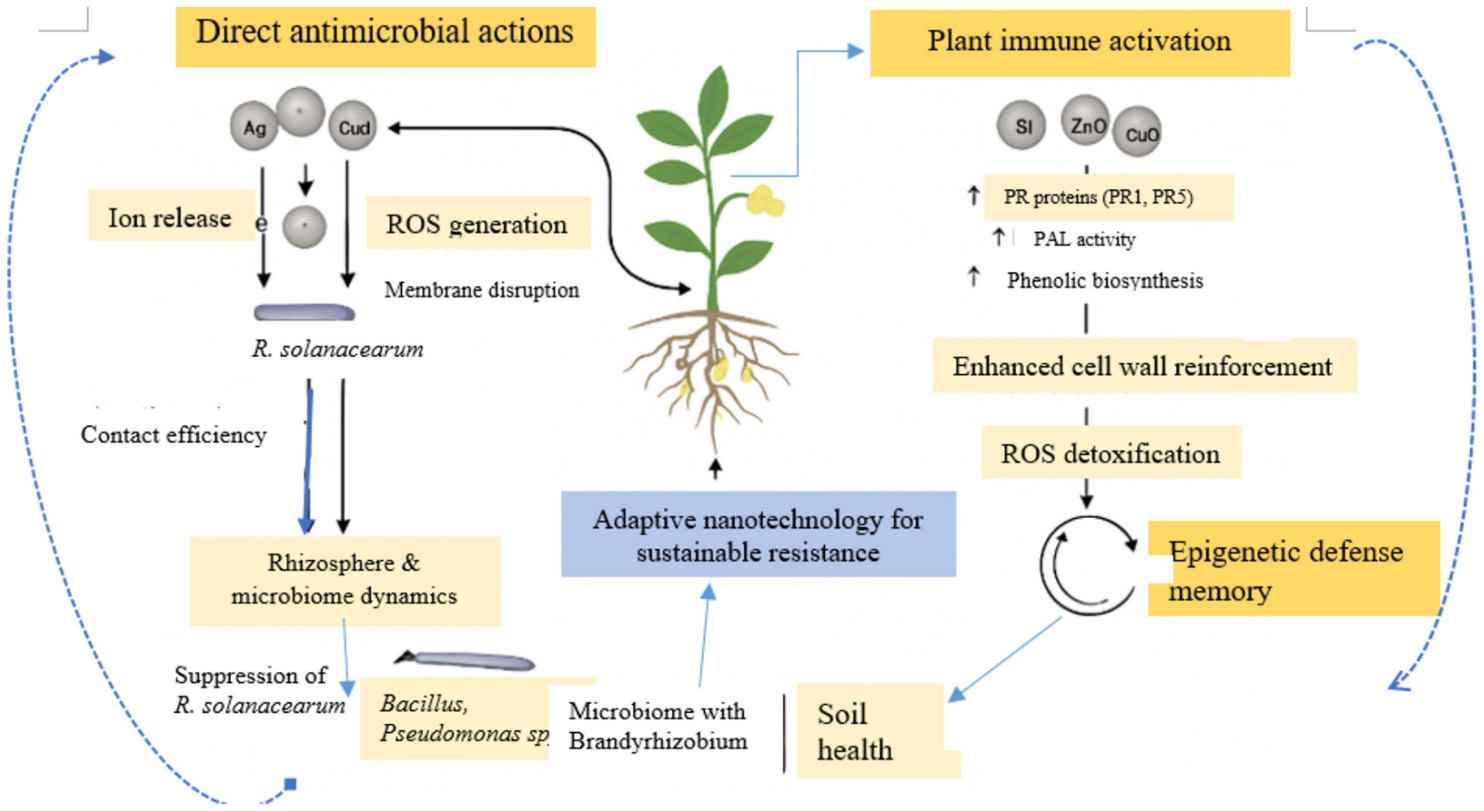
NP mechanisms in peanut *Ralstonia solanacearum* defense.

From a critical perspective, the dual functionality of NPs as both direct antimicrobial agents and elicitors of plant immune responses represents a promising yet underexplored frontier in plant pathology (Carrillo-Lopez et al., 2024). Despite encouraging evidence from model and solanaceous crops, comprehensive molecular and transcriptomic analyses in peanut are still lacking. Thus, understanding the precise signaling pathways, gene networks, and metabolic responses involved in NP–plant–pathogen interactions is essential to optimize their use. The figure (Figure 2) depicts how NPs exert dual effects by directly killing *R. solanacearum* and activating peanut immune pathways. It also shows rhizosphere modulation that supports beneficial microbes and enhances soil health for sustainable resistance.

## 4 Pathogen biology and delivery challenges in vascular wilt systems

A central challenge in the control of bacterial wilt lies in the localization of *R. solanacearum* within the host plant. Once infection occurs, the pathogen rapidly colonizes the root cortex and invades the xylem vessels, leading to systemic spread and vascular blockage (Mori et al., 2016; Xue et al., 2020). This internal localization makes conventional and chemical treatments largely ineffective, as most antimicrobial agents cannot penetrate deep into vascular tissues where bacterial populations reside (Wang Z. et al., 2023). Achieving effective delivery of active compounds to these protected infection sites is, therefore, one of the most critical barriers to managing bacterial wilt in peanut.

Foliar-applied NPs can be absorbed through stomata or cuticular pathways and subsequently translocated via the phloem to other plant tissues, including roots (Seleiman et al., 2020).

Additionally, foliar delivery has been shown to stimulate systemic acquired resistance (SAR) and induced systemic resistance (ISR), enhancing the plant’s defensive capacity against root-infecting pathogens (Zhang et al., 2022). Nanocomposites containing micronutrients or antimicrobial agents may upregulate defense-related enzymes, phenolic compounds, and pathogenesis-related proteins, thereby reducing pathogen colonization even in belowground tissues (Noman et al., 2023).

Furthermore, foliar application minimizes direct soil exposure, potentially reducing environmental accumulation and non-target effects, making it an attractive complementary strategy to soil or seed-based delivery systems (Ding et al., 2023).

However, the transport and bioavailability of NPs within the soil–plant continuum are hindered by several physicochemical and biological factors (Gao et al., 2023). In soil, NPs tend to aggregate or adsorb onto organic matter and clay particles, significantly reducing their mobility and uptake by roots (He et al., 2019; Strekalovskaya et al., 2024). Once in contact with plant tissues, additional barriers such as the cell wall’s pore size and cuticular layers further restrict NP translocation to vascular tissues (Wu and Li, 2022). The term “cell wall pore size” refers to the effective size exclusion limit of the plant cell wall matrix, which is determined by the spacing between cellulose microfibrils and associated polysaccharides (Carpita et al., 1979). This pore size typically ranges from 5 to 20 nm, influencing the passive movement of NPs into the apoplast.

The term “endodermis Casparian” is a specialized root cell layer characterized by the Casparian strip, which regulates the apoplastic movement of water and solutes into the vascular system (Wang et al., 2019). Transport across the endodermis requires symplastic movement via membrane transporters or plasmodesmata. Hence, the combined effects of these barriers limit both the concentration and uniformity of NPs reaching *R. solanacearum* colonization sites, thus reducing antimicrobial efficacy.

Consequently, to overcome these limitations, researchers have proposed several strategic approaches for optimizing NP delivery. The use of ultra-small NPs (<20 nm) can enhance root penetration and systemic movement within plant tissues (Gao et al., 2023). For instance, root-targeted formulations, including hydrogels, nanoemulsions, and nanocomposite carriers, offer the advantage of localized and sustained NP release in the rhizosphere, aligning delivery with pathogen entry points (Davidson et al., 2013). Furthermore, synchronizing NP application with key stages of pathogen invasion such as initial root infection may further increase treatment effectiveness while minimizing resource waste and environmental exposure (Nile et al., 2022; Lee and Kasote, 2024). Thus, these strategies reflect an evolving emphasis on precision delivery and plant-compatible formulation design in nanotechnology-based plant protection.

**Figure 3:**
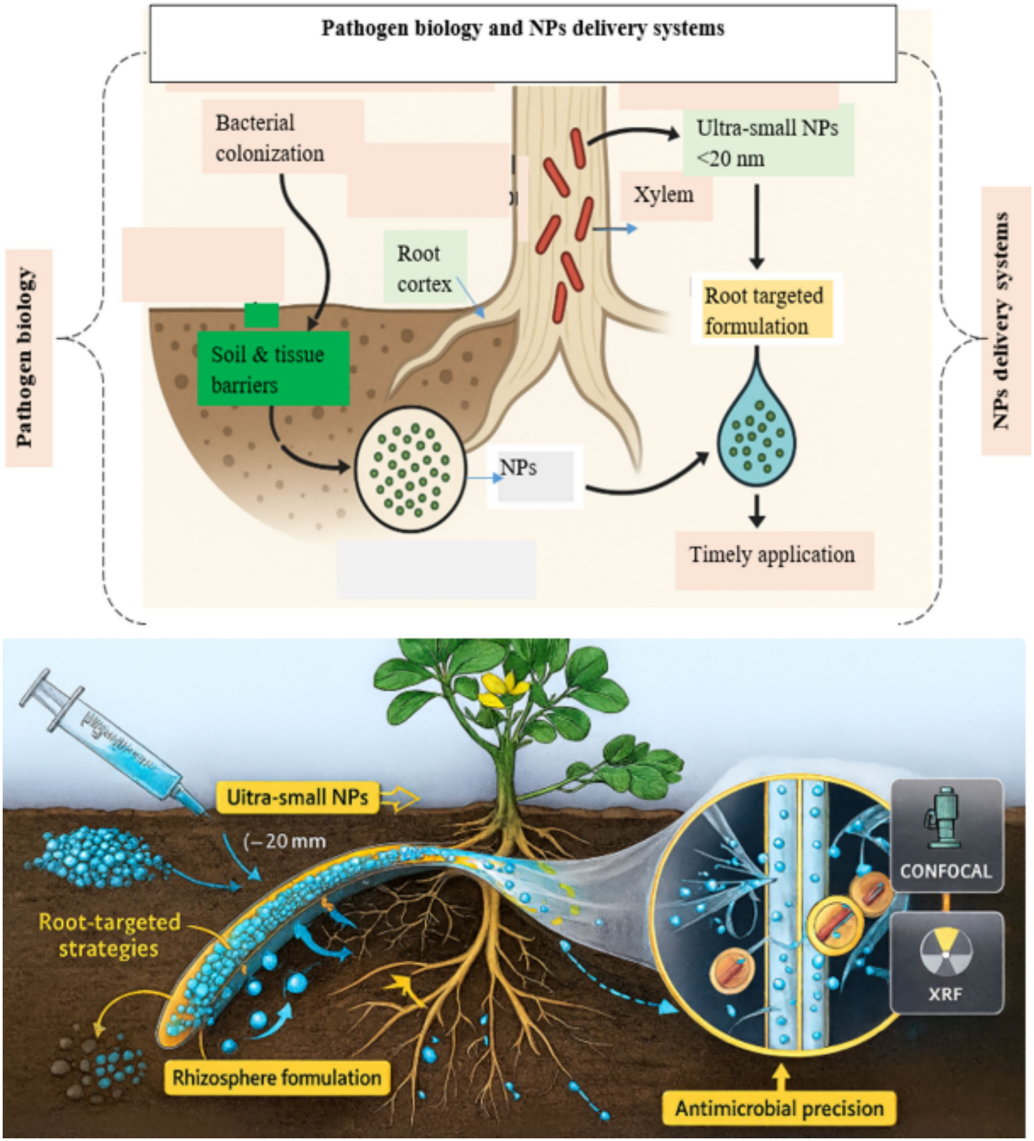
Pathogen biology and NP delivery for managing peanut bacterial wilt.

However, despite these promising advances, a critical knowledge gap persists regarding the *in vivo* behavior and distribution of NPs within peanut tissues. Real-time tracking of NP movement, accumulation, and interaction within the vascular system remains largely unexplored. In addition, advanced imaging techniques such as confocal microscopy, synchrotron-based X-ray fluorescence, and isotopic tracing should also be prioritized to elucidate NP transport dynamics. Overall, NPs face multiple soil and tissue barriers before reaching *R. solanacearum* infection sites in peanut vascular tissues. Ultra-small, root-targeted, and smartly timed NP formulations improve penetration, mobility, and antimicrobial precision (Figure 3).

## 5 Environmental fate and ecotoxicological considerations

While NP-based interventions hold promise for managing bacterial wilt, their long-term soil and ecosystem impacts require careful evaluation. Repeated application of NPs may inadvertently affect non-target soil microorganisms that are essential for nutrient cycling and plant health (Das et al., 2025). Symbiotic microbes such as *Rhizobium* spp., responsible for nitrogen fixation, and arbuscular mycorrhizal fungi that enhance nutrient uptake, are particularly vulnerable to metallic NPs (Fan et al., 2014). Disruptions in these microbial communities can impair soil fertility, alter enzymatic activities related to carbon and nitrogen metabolism, and reduce the ecological resilience of peanut production systems (Wu et al., 2023). Hence, understanding the ecological thresholds of NP exposure is critical for ensuring that disease control does not compromise long-term soil health.

Another emerging concern is the bioaccumulation and residue formation of NPs in edible plant parts, particularly peanut kernels. Due to their small size and potential for systemic movement, certain NPs can translocate through plant tissues and accumulate in reproductive organs (Djanaguiraman et al., 2024; Rico et al., 2011). This raises legitimate food safety concerns, as chronic human or animal exposure to residual NPs could pose toxicological risks. The persistence and transformation of NPs in harvested products necessitate stringent monitoring and assessment frameworks before large-scale agricultural deployment (Atanda et al., 2025).

Current evidence indicates that NP accumulation in reproductive organs (flowers, fruits, seeds) is highly dependent on NP size, solubility, surface charge, and application route (Minello et al., 2025). Although some studies report limited translocation of ultra-small or highly mobile NPs to reproductive tissues, most engineered NPs tend to accumulate predominantly in roots, stems, and leaves.

More importantly, several investigations suggest that NPs larger than the size exclusion limits of vascular tissues show restricted movement into reproductive organs (Gao et al., 2023; Sembada and Lenggoro, 2024). This limited accumulation is often considered advantageous from a food safety perspective. However, the existing data remain fragmented, and systematic studies on NP fate during reproductive development are still scarce.

**Figure 4:**
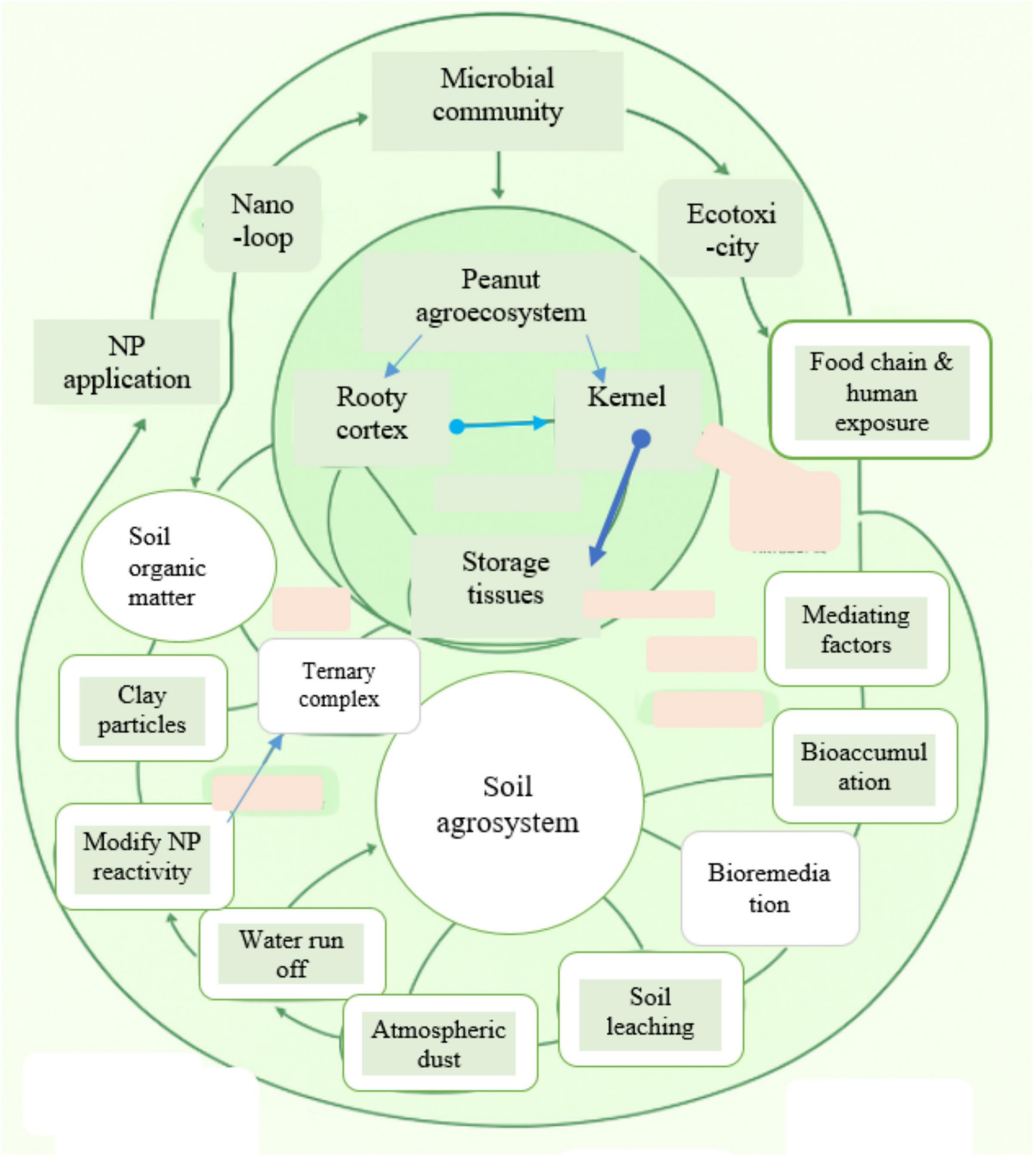
Fate and transport of NPs in peanut agroecosystem and soil agrosystem.

Moreover, environmental transformation processes can significantly alter NP behavior and toxicity in the agricultural environment (Islam, 2025). Upon entering to soil or water systems, NPs may undergo oxidation, dissolution, aggregation with organic and inorganic compounds (Haydar et al., 2025). These transformations can modify their surface chemistry, potentially reducing or enhancing their reactivity and toxicity (Figure 4). For instance, the dissolution of metal-based NPs may release ionic species with higher toxicity than the original particles, while complexation with humic substances could mitigate harmful effects (Islam, 2025).

From a critical standpoint, much of the current research on NP biosafety remains short-term and lacks ecological depth. Most studies emphasize acute toxicity under controlled conditions, often neglecting chronic, low-dose exposures and the intricate interactions among soil, peanut plants, and associated microbial communities (Muzammil et al., 2023; Wang Q. et al., 2023). These oversights hinder the development of comprehensive risk assessment models customized to legume-based systems, which differ significantly from cereals or solanaceous crops in their root structure and symbiotic relationships (Shahroz et al., 2024). Accordingly, establishing standardized, crop-specific risk assessment protocols encompassing both environmental and food safety dimensions is urgently needed to ensure that NP applications in peanut cultivation are both effective and ecologically sustainable (Atanda et al., 2025).

Phytotoxicity assessment is essential to distinguish antimicrobial efficacy from unintended plant damage and the key validation methods include morphological analyses: root length, shoot biomass, leaf area, and visual symptom scoring; physiological indicators: chlorophyll content, photosystem II efficiency (Fv/Fm), and gas exchange parameters; biochemical markers: ROS levels, antioxidant enzyme activity, and lipid peroxidation; histological and cytological assays: membrane integrity staining and tissue ultrastructure analysis, and microbial interaction assays: evaluating whether NPs selectively inhibit pathogens without disrupting beneficial plant-associated microbiota (Gao et al., 2023; Djanaguiraman et al., 2024; Tarrahi et al., 2021).

These integrated approaches are particularly important in plant–bacteria systems to ensure that disease suppression results from targeted antimicrobial action rather than generalized toxicity.

## 6 Formulation, scale-up, and field application challenges

**Figure 5:**
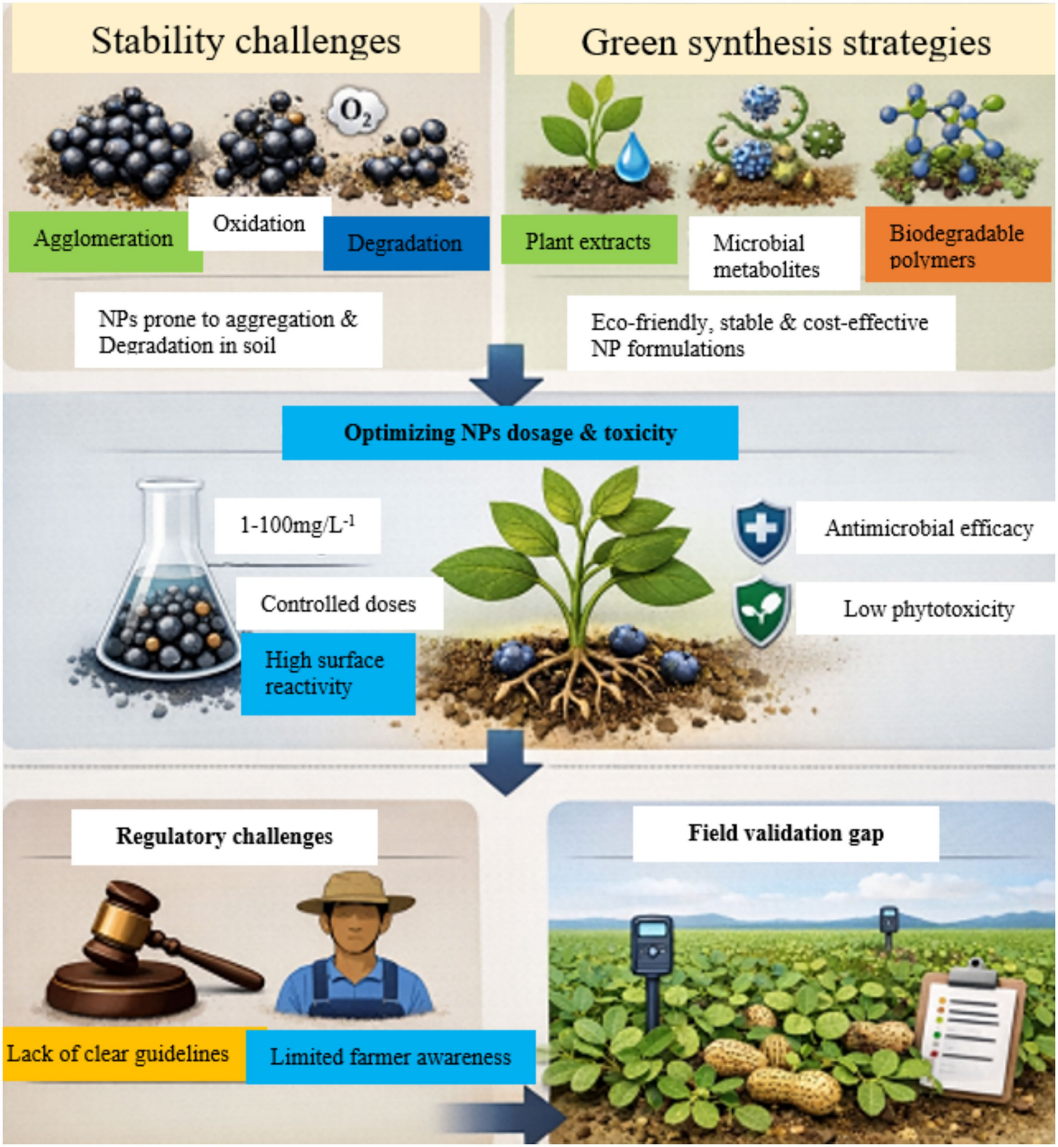
Formulation, scale-up, and field application challenges.

Despite notable laboratory successes, the translation of NP technologies into field-ready formulations faces several unresolved challenges (Figure 5). The figure indicates the interconnection between NP stability, green synthesis, dose optimization, and regulatory constraints in achieving effective and safe disease management. Ensuring NP stability under variable environmental conditions remains a major technical barrier, as particles are prone to agglomeration, oxidation, or premature degradation in soil matrices (Khanna et al., 2021). These issues compromise controlled release and reduce antimicrobial efficacy over time. To overcome such limitations, novel formulation strategies are focusing on biogenic synthesis routes utilizing plant extracts, microbial metabolites, or biodegradable polymers to produce NPs with inherent stability, uniform morphology, and eco-compatible surfaces (Patil and Chandrasekaran, 2020; Geszke-Moritz and Moritz, 2024). Such “green synthesis” approaches not only minimize the need for hazardous reducing agents but also lower production costs, thereby improving scalability and environmental safety (Osman et al., 2024).

NP dosages used for antimicrobial applications in plants typically range from 1 to 100 mg/L^−1^, depending on NP composition, size, surface functionalization, and plant species (Masood et al., 2024). Lower concentrations are generally sufficient due to the high surface reactivity and enhanced bioavailability of nanomaterials (Mgadi et al., 2024).

Phytotoxicity is closely linked to dose, exposure duration, and NP chemistry. At optimal concentrations, NPs can exert antimicrobial effects while maintaining cellular redox balance, membrane integrity, and photosynthetic efficiency in plants (Ghorbani et al., 2024). Surface modifications (e.g., polymer coatings or biogenic synthesis routes) further reduce toxicity by limiting excessive metal ion release or reactive oxygen species (ROS) generation (García-Torra et al., 2021).

Therefore, dose optimization is essential to ensure antimicrobial efficacy without impairing plant growth or physiology.

Also important are the regulatory and translational dimensions of NP adoption in peanut disease management. The absence of clearly defined regulatory frameworks for “nano-enabled pesticides” continues to impede commercialization and field deployment (Wei et al., 2025; Ullah et al., 2024). Current safety and efficacy testing protocols are also designed for conventional agrochemicals and fail to capture NP-specific behaviors such as bio-distribution (Eghbalinejad et al., 2024). This regulatory ambiguity, compounded by limited farmer awareness and inadequate technological infrastructure in developing regions, hinders adoption at scale (Gelaye, 2025). Critically, field-based validation across diverse peanut-growing environments remains scarce, resulting in a gap between experimental promise and practical feasibility.

## 7 Comparative evaluation and future directions

NP-mediated antimicrobial delivery offers clear advantages over conventional chemical sprays, including targeted action, longer persistence, and reduced environmental impact (Izuafa et al., 2025). However, field validation remains limited compared to biocontrol agents and resistant peanut cultivars, which have demonstrated consistent performance. Integrating NPs with biocontrol organisms (*Pseudomonas*, *Bacillus*) or resistant varieties could provide synergistic benefits, combining immediate pathogen suppression with durable and eco-friendly protection (Upadhayay et al., 2023).

**Figure 6:**
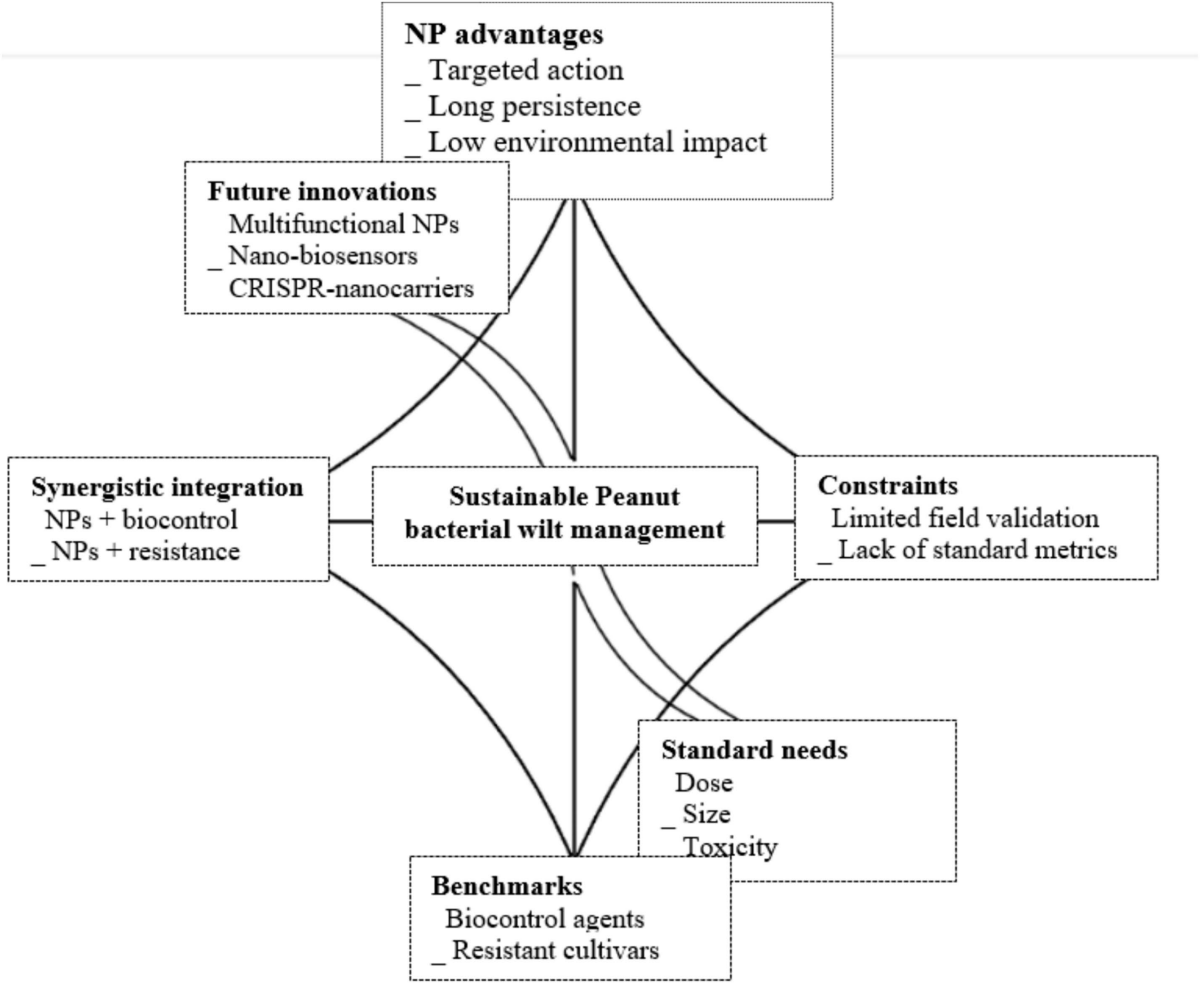
Comparative evaluation and future development of NP-mediated disease management in peanut.

Future innovations include multifunctional NPs combining antimicrobial, elicitor, and sensing roles, nano-biosensors for early pathogen detection, and CRISPR–nanocarrier systems for targeted resistance gene delivery (Figure 6). The lack of standardized evaluation metrics including dose, particle size, and toxicity endpoints hindering cross-study comparison and regulatory clarity are also a critical challenge. Consequently, addressing these gaps through unified testing, field validation, and interdisciplinary research will be key to translating nanotechnology into safe and effective strategies for sustainable peanut cultivation.

## 8 Conclusion

In conclusion, this critical review discusses the potential of NP-based antimicrobial delivery as a precise and sustainable strategy for managing bacterial wilt in peanut. NPs offer controlled release, vascular targeting, and the ability to prime plant defenses, complementing conventional approaches such as resistant cultivars and biocontrol agents. However, challenges persist in fully understanding NP–plant–pathogen interactions, assessing environmental and food-safety risks, and scaling laboratory innovations to field conditions. Formulation stability, regulatory gaps, and limited agroecological validation further constrain practical adoption.

Future research should focus on mechanistic insights, eco-safety evaluations, and standardized field trials to optimize NP design and application. Integrating nanotechnology within holistic disease-management frameworks rather than using it as a standalone solution can enhance efficacy, reduce chemical inputs, and support sustainable peanut production.
